# Intraspecific variability in seed mass, germination, and seedling growth in the narrow endemic *Iberodes littoralis* subsp. *gallaecica*

**DOI:** 10.1093/aobpla/plag017

**Published:** 2026-04-06

**Authors:** Julia Sánchez Vilas, Josefina G Campoy, Mariana Fiuza, Rubén Retuerto

**Affiliations:** School of Biosciences, Cardiff University, Sir Martin Evans Building, Cardiff CF10 3AX, United Kingdom; CRETUS, Department of Functional Biology (Area of Ecology), Universidade de Santiago de Compostela, Santiago de Compostela, Spain; Centre for Functional Ecology, Associate Laboratory TERRA, Department of Life Sciences, University of Coimbra, Coimbra 3000-456, Portugal; Department of Functional Biology (Area of Ecology), Universidade de Santiago de Compostela, Santiago de Compostela, Spain; Department of Functional Biology (Area of Ecology), Universidade de Santiago de Compostela, Santiago de Compostela, Spain

**Keywords:** endemism, seed ecology, restoration, coastal dunes, population variability

## Abstract

The study of seed trait variation is essential for developing effective conservation strategies, understanding plant recruitment, and predicting the long-term persistence of endemic/rare plant species under environmental change. This study examines the seed ecology of *Iberodes littoralis* subsp. *gallaecica*, a narrow endemic to coastal dune systems in north-western Spain. Seeds collected from five populations over 7 years were grown in a glasshouse to investigate variation in seed mass, germination rate, time to germination, and seedling relative growth rate (RGR). To evaluate the role of resource availability, seeds were sown in two substrate types: dune sand alone and mixed with compost. Seed traits varied significantly among years and populations. Overall, older seeds germinated less and took longer to germinate than younger seeds. Heavier seeds had greater germination percentages, but the effect of seed mass on time to germination differed among years. Seeds produced under higher maternal rainfall were lighter, which in turn influenced germination timing and seedling RGR, indicating an indirect effect of maternal environment on seed performance. Germination rates were lower in the substrate amended with compost, but those seedlings had a higher RGR. These results suggest that using fresh seeds maximizes restoration success, and when fresh material is unavailable, the largest seeds should be selected. While fertilisation can enhance early seedling growth, it may not improve germination success. Our findings highlight the complex interplay among seed age, seed mass, population differences and substrate conditions in shaping germination and early growth of this narrow endemic.

## Teaser

Seed traits strongly influence plant recruitment and conservation outcomes in rare species. We show that in a coastal dune endemic, seed age, seed size, origin, and substrate jointly shape germination and early growth. Fresh and larger seeds perform best, while nutrient enrichment enhances growth but reduces germination. These findings highlight the need to consider multiple interacting factors when selecting seeds for restoration under changing environmental conditions.

## Introduction

Coastal ecosystems are under high rates of anthropogenic pressure due to population growth and industrialisation and are also highly vulnerable to the effects of climate change (Loarie et al. 2008). Climate-induced changes are expected to enhance the magnitude of existing natural and anthropogenic stress factors (Frosini et al. 2012), increasing the threats to plant species that inhabit coastal dunes (Frey et al. 2016). Fixed coastal dunes with herbaceous vegetation (grey dunes) are characterised by high environmental heterogeneity at the geomorphological and microclimatic levels, which favours the existence of a large variety of species and promotes the presence of specialised and endemic species adapted to specific dune conditions (Lomba et al. 2008). Such endemic species are at risk of declining due to their limited dispersal ability and specialised habitat requirements (Coelho et al. 2020, Manes et al. 2021). Beyond species loss, the degradation of coastal habitats would negatively impact the critical socioeconomic and ecological services they provide, including their pivotal role as blue carbon stocks (Beaumont et al. 2014, Del Vecchio et al. 2022). Therefore, the conservation of coastal ecosystems is vital, including the preservation of native vegetation (Lomba et al. 2008, Del Vecchio et al. 2018).

In this context, studying variation in seed traits—such as seed mass, viability, and germination—is crucial for developing effective conservation strategies, understanding plant recruitment and predicting the long-term persistence of endemic or rare plant species in the face of environmental change (Rashid et al. 2023). In coastal dunes—characterised as nutrient-poor, water-limited, and spatially heterogeneous environments—seed mass is expected to play a central ecological role. Larger seeds generally exhibit higher germination success and support faster early seedling growth through greater internal reserves (Moles and Westoby 2004, Fenner and Thompson 2005). These reserves can also buffer against drought and nutrient limitation, common stressors in dune systems (Leishman et al. 2000, Vaughton and Ramsey 2001). In contrast, smaller seeds may persist longer in the soil, contributing to the formation of soil seed banks (Fenner and Thompson 2005, Genna et al. 2020), although empirical patterns may vary across species (Karrenberg and Suter 2003, Gioria et al. 2020, Endrédi et al. 2023, Huayta-Hinojosa et al. 2025). Thus, seed mass variation within a species may reflect adaptive responses to the environmental variability characteristic of dune ecosystems and may play a crucial role in adaptation to changing conditions (Razzaque et al. 2023).

Importantly, the effect of seed mass on seed traits such as germination, establishment, and viability is likely modulated by external environmental factors, including climatic conditions and the substrate in which seeds germinate (Pawłowski et al. 2024, Homberger et al. 2025). Temperature, rainfall, and other climate variables can influence seed provisioning and affect subsequent germination success (Pawłowski et al. 2024). In addition, substrate characteristics also play a critical role in germination success and early seedling establishment in dunes, where sandy soils are nutrient-poor and have low water retention, which can lead to poor seedling survival (Homberger et al. 2025). For restoration purposes, amending dune sand with organic materials may improve water-holding capacity and nutrient availability; however, nutrient-rich soils can also alter water retention and microbial communities in ways that may negatively affect germination rates (Chee-Sanford et al. 2006).

Populations of endemic species, especially those with a narrow and fragmented distribution, may experience unique selective pressures, leading to local adaptations in seed traits that are fine-tuned to local environmental conditions (Cochrane et al. 2015). Investigating population-level variation in seed traits and seedling performance under different environmental and substrate contexts can provide valuable insights for plant conservation and restoration. Management of rare plants often prioritizes maintaining genetic diversity and successful recruitment, and these efforts will be supported by information on juvenile plant demography from different populations and different growing conditions (Merritt and Dixon 2011, Johnston et al. 2023).

In this study, we aim to improve our understanding of the seed ecology of *Iberodes littoralis* subsp. *gallaecica* (Laínz) M. Serrano, R. Carbajal & S. Ortiz (=*Omphalodes littoralis* subsp. *gallaecica* M. Laínz) by investigating variability in key seed traits across populations and years. *I. littoralis* subsp. *gallaecica* is a small annual herb belonging to the Boraginaceae family, which is endemic to coastal dune systems in the northwest of the Iberian Peninsula (Serrano and Carbajal 2004, Buján 2005). The species is endangered and facing a steady decline due to threats to its fragile habitat, resulting in a highly fragmented and narrow distribution (Alvite et al. 2025). In particular, here, we investigate how seed provenance and collection year influence seed mass, germination rate, and time to germination, as well as seedling growth. In addition, to better understand how resource availability influences these effects, we conducted germination experiments using two different substrate types: dune sand alone and a mixture of dune sand and compost.

## Materials and methods

### Study species

**Figure 1:**
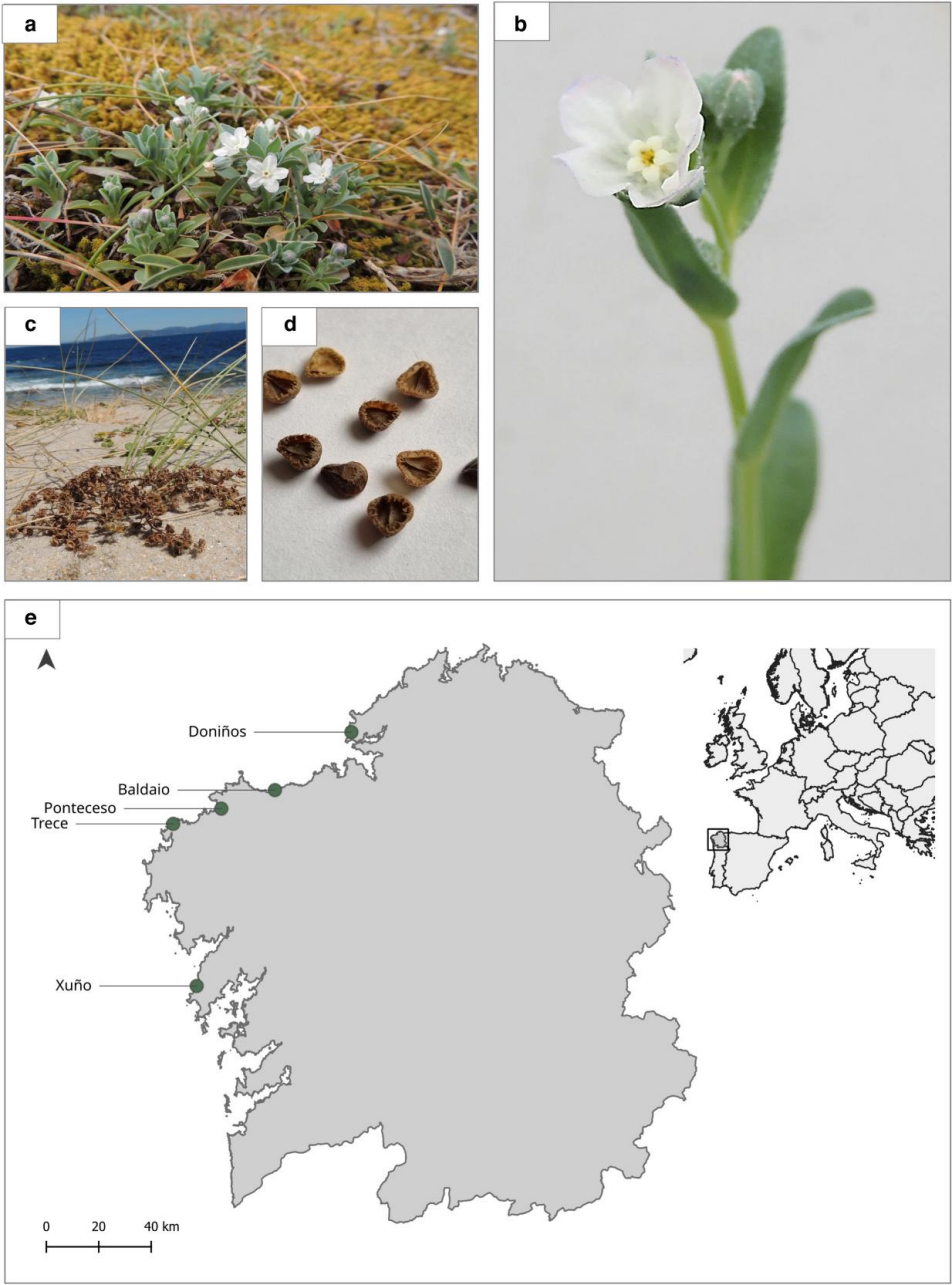
Representative images of *Iberodes littoralis* subsp. *gallaecica* and distribution of the studied populations in coastal dune systems of the north-western Iberian Peninsula. (a) Individual growing in grey dunes during the flowering stage, (b) flower detail, (c) plant bearing mature seeds in its natural habitat, (d) detail of mature seeds, and (e) map of Galicia (NW Spain) showing the five studied populations; the inset indicates the position of the study area within Europe. © Josefina G. Campoy.

*Iberodes littoralis* subsp. *gallaecica* (Boraginaceae) is an endemic and rare annual herb (total occupancy <100 000 m^2^) that only grows in a few coastal dune systems in NW Spain (Alvite et al. 2025). Previously classified as *Omphalodes littoralis* subsp. *gallaecica*, it was reclassified under the genus *Iberodes* following taxonomic revisions (Serrano et al. 2016). *I. littoralis* subsp. *gallaecica* is catalogued as ‘endangered’ by the IUCN (Serrano and Carbajal 2006), and by both the Spanish and Galician Catalogues of Threatened Species (Serrano and Carbajal 2004, DOG 2007). Additionally, this plant is listed as a priority species in the EU Habitats Directive (92/43/EEC, Annex II), and its habitat is considered as a Site of Community Importance (SCI) within the Natura 2000 network. It has a small size of up to 13 cm in height (Serrano and Carbajal 2004). Germination takes place in November–December, with flowering beginning in April and lasting until May (Serrano and Carbajal 2004). Flowers are hermaphroditic and last less than 3 days (Buján 2005). They are self-compatible, and autogamy has been suggested as the most probable mechanism of reproduction (Serrano and Carbajal 2004). Each fruit develops four seeds that are mainly dispersed by exozoochory thanks to specialised hooks that easily attach to the animal's fur (Serrano and Carbajal 2004). Representative images of the study species and its habitat are provided in Fig. 1a–d.

### Seed collection and experimental design

The seeds used in the study were obtained from collections spanning over seven non-consecutive years (2010, 2013, 2016, 2017, 2018, 2020, 2021), in five populations located in coastal dune systems of the north-western Iberian Peninsula (Fig. 1e): Baldaio, Doniños, Ponteceso, Trece, and Xuño. Seeds were collected between May and June of the corresponding years, from at least 25 healthy individuals from each population. Seeds were stored under controlled dry conditions at room temperature until the beginning of the experiment, as vernalization requirements for *I. littoralis* subsp. *gallaecica* are unknown and cold stratification may potentially alter natural germination timing. Of the total number of seeds collected, 80 were randomly selected from each population and year, making 2800 seeds weighed individually with a precision balance (Mettler Toledo XP26, ±0.001 mg).

Seeds were sown in 40 trays, each consisting of 70 cells with a volume of 61 ml per cell. Seeds were sown individually, with each seed placed in its own cell. In each tray, two seeds from each population and year combination were sown (total *N* = 2 seeds × 5 populations × 7 years × 40 trays = 2800 seeds). Half of the trays were filled with beach sand, while the other half were filled with a mixture (2:1) of compost and beach sand. Trays were arranged systematically on the glasshouse benches to minimise positional effects. Seeds were not pre-treated before sowing, as the aim was to evaluate their natural germination behaviour. The experiment was carried out from January to March 2022 in the glasshouse facilities at the Faculty of Biology (University of Santiago de Compostela, Spain), without supplemental lighting or temperature. The plants were bottom watered with tap water when needed.

### Climatic variables

To assess the potential influence of climatic factors on seed and seedling traits, we characterised the climatic conditions at the five locations where the seeds were collected. Given that in natural populations seedlings begin to emerge in November and reach senescence by early June of the following year, we considered the monthly mean temperatures and precipitation levels for the months covering the species’ growing season: October, November, and December of the previous year, as well as from January to May of the seed production year. Climatic data were obtained from the nearest meteorological stations to each collection site, provided by MeteoGalicia (http://www.meteogalicia.es).

### Seed and seedling traits measured

For each seed, germination status was recorded—except on the weekend—daily as 0 (no germination) or 1 (cotyledons emerged from the seed and became visible above the substrate). Germination started on the 14th of January 2022, 10 days after sowing, and the last recorded germination occurred on 3rd March 2022, 58 days after sowing. Time to germination was recorded as the number of days taken from sowing until germination for each individual seed. Plants were harvested on 1st June 2022, dried in an oven at 40°C until reaching a constant mass, and their dry mass was measured. The relative growth rate (RGR) was calculated as: (ln (final plant mass) − ln (seed mass))/days (from germination to harvest).

### Statistical analyses

#### Climatic variables

To reduce dimensionality and minimize collinearity among predictors, climatic variables were summarized as mean temperature and total precipitation for two biologically relevant periods: pre-germination and post-germination. The pre-germination period ranged from October to December of the year before seeds were collected, when seeds were primarily buried in the soil; and post-germination period ranged from January to May of the collection year, when seedlings grew, flowered, and produced the seeds we collected.

#### Seed mass

Because climatic data were identical for all seeds within each population and year combination, climatic variables were confounded with population and year and thus could not be included together as fixed effects in the same model. To account for this structure, we conducted two complementary analyses: First, we used a linear model (lm) to test the effects of population and year of collection and their interaction on the seed mass of *I. littoralis* subsp. *gallaecica*.

Second, to examine the effects of the climate variables (total precipitation and mean temperature in the pre- and post-germination periods) on seed mass, we ran a linear mixed-effects model (LMM) from the lme4 package (Bates et al. 2011). A full LMM included the summarised climatic variables as fixed effects, with random effects for population and year of collection.

Fitted linear and mixed models were validated by inspecting the residual plots using the DHARMa package (Hartig 2022). The significance of fixed effects was assessed using Type III sums of squares with ‘Anova()’ from the ‘car’ package (Fox 2009) for linear models and with ‘anova()’ from the ‘lmerTest’ package for linear mixed-effects models (Kuznetsova et al. 2017). When significant differences among more than two groups were found, pairwise comparisons were carried out using ‘emmeans()’ with Bonferroni adjustment (Lenth 2025). The significance threshold (*α*) was set to 0.05 for all statistical tests reported here and elsewhere in the manuscript. The package ‘visreg’ was used to allow the visualisation of the relationship between seed mass with a given predictor while holding all other variables constant (Breheny and Burchett 2017). Boxplots showing the distribution of seed mass across populations and years were created using the ‘ggplot2’ package in R (Wickham 2016).

#### Germination

Germination dynamics were analysed using time-to-event methods based on Cox proportional hazard models, which allow to evaluate both the timing of germination (days to germination) and the cumulative proportion of seeds germinated over time (germination status: germinated vs. censored). Prior to modelling, years with no or minimal germination (2010 and 2013) were removed to ensure stable and unbiased estimates. We used mixed-effects Cox models fitted with the ‘coxme’ package (Therneau 2024a), which are appropriate to germination studies because they handle right-censored observations arising from seeds that fail to germinate (McNair et al. 2012). Model assumptions were evaluated using Schoenfeld residual diagnostics using the ‘survival’ package (Therneau 2024b).

As above, to account for the confounding effect between population and year with climatic variables, we ran two complementary models. The first included the fixed effects of seed mass, population (Baldaio, Doniños, Ponteceso, Xuño, Trece), year of collection (2016, 2017, 2018, 2020, 2021) and substrate (dune sand, dune sand + compost), with a random intercept for tray. We focused on a model that included only the second-order interactions, as there was not enough statistical power for the higher-order interactions due to a lack of/minimal germination in some of the populations per combination of year and soil. The second model examining climatic influences, included the fixed effects of seed mass, substrate, and pre-germination and post-germination climate variables, and the random intercepts for tray, population, and year. Second order interaction terms of climatic variables with seed mass and substrate were initially considered but ultimately removed based on Akaike information criterion (AIC) model selection to focus on main effects (Farooq and Karami 2019). Significance of fixed effects in ‘coxme’ models was assessed using likelihood ratio tests (LRTs) comparing the full model to reduced models excluding the term of interest.

To visualise the significant effects, predicted cumulative germination curves were generated using ‘survfit()’ from the ‘survival’ package (Therneau 2024b), with all non-focal predictors held constant. Cumulative germination was computed from the model-estimated survival curves as 1 − *S*(*t*), corresponding to the proportion of seeds germinated by each time point.

#### Relative growth rate

To test the effects of individual seed mass, population of origin and age on the RGR of the seedlings of *I. littoralis*, we used a linear mixed effects model (lmer). RGR was used as the response variable and we added population (Baldaio, Doniños, Ponteceso, Xuño, Trece), year of collection (2016, 2017, 2018, 2020, 2021) and substrate (dune sand, dune sand + compost) as fixed factors, and tray was added as a blocking random factor. As above, we focused on a model that included only the second-order interactions, due to insufficient statistical power for the higher-order interactions (Supplementary Table S1). However, given the limited sample size relative to the high number of parameters, the model was still over-parameterized. To obtain a more robust and interpretable model, we simplified the fixed-effect structure using the ‘step()’ function, guided by changes in AIC and likelihood ratio tests (Venables and Ripley 2002). A second model was also run to examine the influence of the climatic variables on RGR, including the fixed effects of substrate, pre-germination and post-germination climate variables, and the random intercepts for tray, population, and year.

Fitted models were validated by inspecting the residual plots using the DHARMa package (Hartig 2022). Significance of the fixed effects was assessed using ‘anova()’ from the ‘lmerTest’ package (Kuznetsova et al. 2017). When significant differences among multiple groups were found, pairwise comparisons were carried out using ‘emmeans()’ with Bonferroni adjustment (Lenth 2025). The package ‘visreg’ was used to allow the visualisation of the relationship between RGR and seed mass while holding all other variables constant (Breheny and Burchett 2017). Boxplots showing the distribution of RGR across populations and years were created using the ‘ggplot2’ package in R (Wickham 2016). All analyses were conducted in R v. 4.2.3 (R Core Team 2022).

## Results

### Seed mass

**Figure 2:**
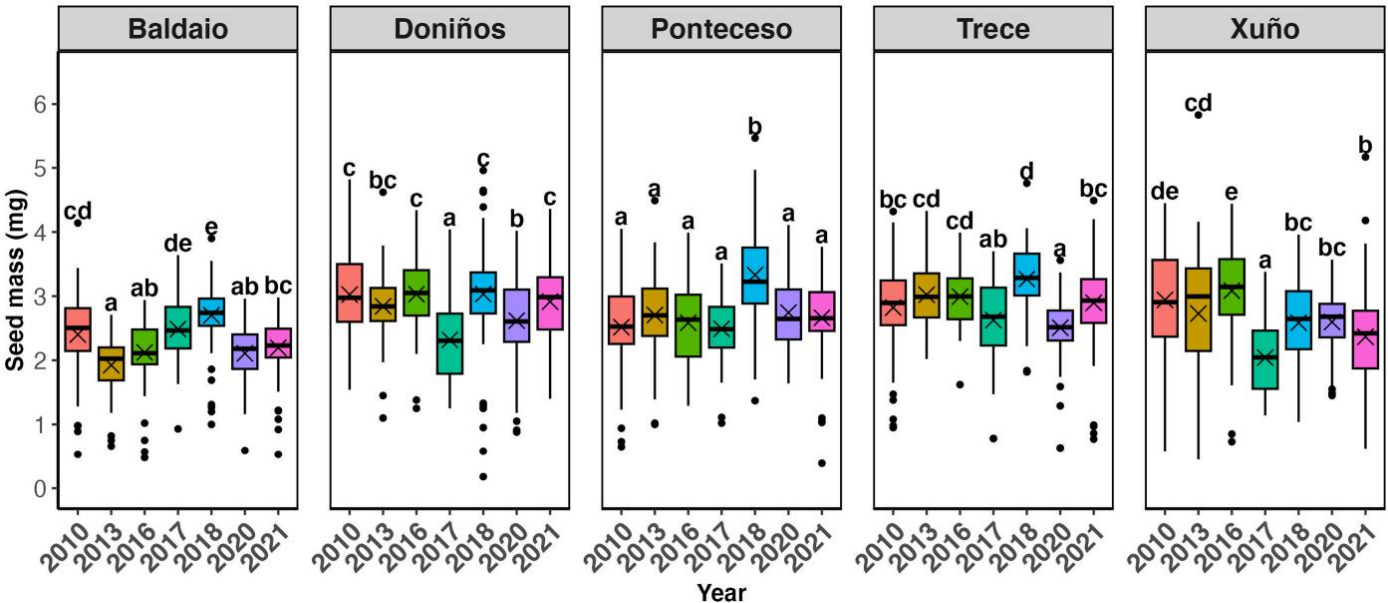
Boxplots showing the distribution of seed mass of *Iberodes littoralis* subsp. *gallaecica* across the five populations studied over seven collection years (*N* = 80). Within each box, horizontal lines denote median values, crosses denote mean values; boxes extend from the 25th to the 75th percentile of each group's distribution of values; whiskers denote adjacent values (i.e. the most extreme values within 1.5 interquartile range of the 25th and 75th percentile of each group); dots denote observations outside the range of adjacent values. Different letters above each box indicate significant differences between years within the same population (*P* < 0.05).

The seed mass differed across populations and years (Pop × year: *P* < 0.001; Table 1). The interaction shows great variability in the seed mass of the populations across time, with some populations remaining fairly constant across years (e.g. Ponteceso), whilst others display more variation (Fig. 2). Overall, averaging across years, populations differed markedly in mean seed mass, with Baldaio producing the smallest seeds (2.28 ± 0.03 mg), Doniños and Trece the largest (2.38 ± 0.03 and 2.88 ± 0.03 mg, respectively), and Ponteceso (2.73 ± 0.03 mg) and Xuño (2.62 ± 0.03) showing intermediate values.

**Table 1:** Results of the linear model to test for the effects of population (Baldaio, Doniños, Ponteceso, Xuño, Trece), year of collection (2010, 2013, 2016, 2017, 2018, 2020, 2021) and their interaction on seed mass.

Explanatory variable	*df*	*F*-value	*P*-value
Population (Pop)	4	83.95	**<0**.**001**
Year of collection (Year)	6	38.46	**<0.001**
Pop × Year	24	12.23	**<0.001**
Error	2765		

df = degrees of freedom. Significant results (*P* < 0.05) are marked in bold.

The linear mixed models revealed that accumulated precipitation during the post-germination period, when the parent seedlings grew and plants produced the study seeds, strongly influenced seed mass (*F*_1,257_ = 9.03, *P* = 0.003). Specifically, higher accumulated precipitation during the post-germination period was associated with lower seed mass (Supplementary Figure S1). Accumulated precipitation during the pre-germination period, and average temperatures during the pre-germination and post-germination periods did not significantly affect seed mass (*P* > 0.05; Table 2).

**Table 2:** Results of the linear mixed effects model to test for the effects of the climatic variables in seed mass.

Explanatory variable	*df*_num_/*df*_den_	*F*-value	*P*-value
Average temperature during the pre-germination period (°C)	1/216	1.98	0.161
Accumulated precipitation during the pre-germination period (mm)	1/559	3.21	0.074
Average temperature during the post-germination period (°C)	1/441	1.1	0.296
Accumulated precipitation during the post-germination period (mm)	1/257	9.03	**0**.**003**

Significance of fixed effects was assessed using Type III sums of squares with Satterthwaite’s approximation for degrees of freedom. *df*_num_ = degrees of freedom of the numerator, *df*_den_ = degrees of freedom of the denominator. Significant results (*P* < 0.05) are marked in bold.

### Germination dynamics

**Figure 3:**
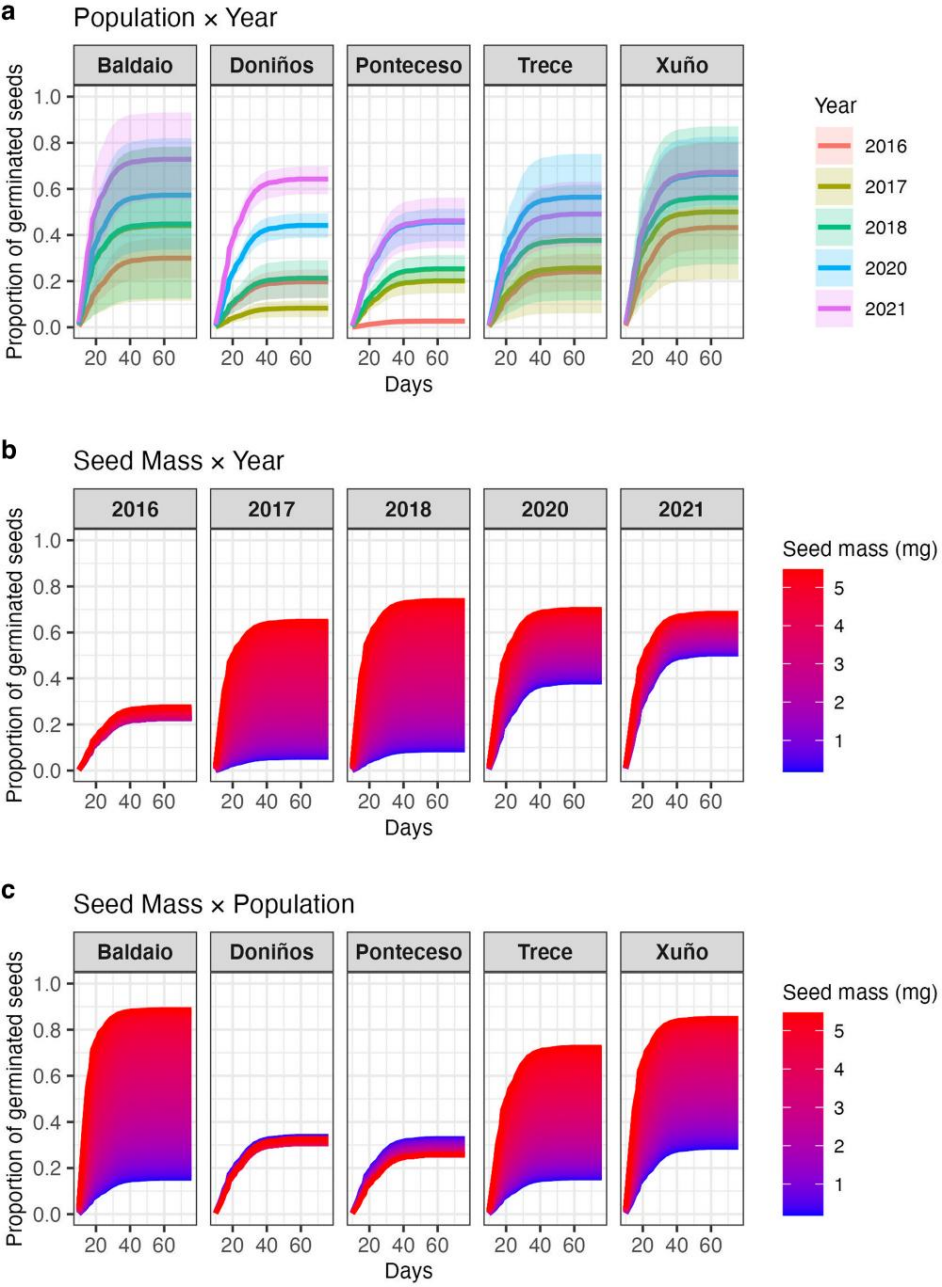
Predicted cumulative germination based on fitted survival models, showing (a) the interacting effects of population and year—with shaded areas representing 95% confidence intervals, (b) the interacting effects of seed mass and year, and (c) the interacting effects of seed mass and population—with colour gradient indicating increasing seed mass (mg). All non-focal predictors were held constant, so plots represent partial effects of the focal predictors on germination dynamics (timing in days and proportion of seeds germinated) of *Iberodes littoralis* subsp. *gallaecica*.

Germination timing and proportion of germinated seeds were affected by age in all populations, with older seeds collected in 2010 not germinating at all, and those collected in 2013 with minimal germination across all populations (not germinating for Ponteceso and Trece, and only one seed germinating for Baldaio, Doniños and Xuño). When analysing data from 2016 onwards, there was variation in the response of each population over time (Population × Year: *χ*^2^ = 60.86, *df* = 16, *P* < 0.0001), with most populations decreasing germination and taking longer to germinate the older the seeds were (Fig. 3a). These differences between older (2016–2018) and younger seeds (2020–2021) were particularly marked in Doniños and Ponteceso (Fig. 3a).

Interestingly, seed mass affected the probability of germination differently across years (Seed Mass × Year: *χ*^2^ = 14.36, *df* = 4, *P* = 0.006). Overall, larger seeds germinated earlier and reached higher cumulative germination than smaller seeds (Fig. 3b). This pattern was more pronounced in 2017 and 2018 and the cumulative germination was the lowest for 2016, where seed mass had little effect on germination dynamics (Fig. 3b). Seed mass also influenced the germination timing and the proportion of germinated seeds depending on the origin (Seed Mass × Pop: *χ*^2^ = 18.21, *df* = 4, *P* = 0.001). For seeds from Baldaio, Trece and Xuño there was a clear trend with larger seeds germinating earlier and reaching higher cumulative germination than smaller seeds. Moreover, these populations reached higher cumulative germination than Doniños and Ponteceso, where seed mass did not affect much the germination variables measured; if anything, it appears they have an opposite trend with the lowest seed mass reached the highest values of cumulative germination (Fig. 3c). Finally, substrate showed a marginal effect that fell short of statistical significance (*χ*^2^ = 17.13, *df* = 10, *P* = 0.072). Other tested interactions were also not significant (all *P* > 0.1, see Table 3).

**Table 3:** Likelihood ratio tests for fixed effects in the Cox mixed-effects model for germination.

Explanatory variable	*χ* ^2^	*df*	*P*-value
Seed mass	52.84	10	**<0**.**0001**
Population (Pop)	173.48	28	**<0.0001**
Year of collection (Year)	318.80	28	**<0.0001**
Substrate (Subs)	17.13	10	0.072
Seed mass × Pop	18.21	4	**0**.**001**
Seed mass × Year	14.36	4	**0**.**006**
Seed mass × Subs	0.68	1	0.409
Pop × Year	60.86	16	**<0.0001**
Pop × Subs	7.70	4	0.103
Year × Subs	0.30	4	0.990

Seed mass (mg), population (Baldaio, Doniños, Ponteceso, Xuño, Trece), year of collection (2016, 2017, 2018, 2020, 2021), and substrate (sand, sand + added compost) were included as fixed effects, together with their second order interactions. Each term was tested by comparing the full model with a reduced model omitting the term of interest. The Chi-squared (*χ*^2^) statistic, degrees of freedom (*df*), and *P*-value are reported. Significant effects (*P* < 0.05) are indicated in bold.

The analysis of the climatic variables found only a marginal effect of the average temperature in the pre-germination period that fell short of significance (*χ*^2^ = 3.36, *df* = 1, *P* = 0.067; Supplementary Figure S2a). Accumulated precipitation in the pre- and post-germination periods, and the average temperature in the post-germination period did not significantly affect germination timing (all *P* > 0.3). The positive effect of seed mass on germination timing and cumulative germination was also corroborated in this analysis (*χ*^2^ = 26.46, *df* = 1, *P* < 0.001). Here, the effect of substrate on germination dynamics was significant (*χ*^2^ = 8.54, *df* = 1, *P* = 0.003), with seeds in sand achieving a higher proportion of germination and germinating earlier than in compost-amended sand (Supplementary Figure S2b).

### Relative growth rate

The RGR of seedlings was affected by the interaction of population and age (Population × Year: *F*_16,642_ = 2.49, *P* = 0.001), and by substrate (Substrate: *F*_1,39_ = 5.89, *P* = 0.020). The RGR varied among populations across years, with no consistent pattern in relation to seed age (Supplementary Figure S3). Baldaio and Ponteceso showed similar RGR across years, whereas Doniños, Trece, and Xuño showed significant differences in RGR across years (Supplementary Figure S3). However, these differences did not follow a consistent directional trend, as intermediate years often showed higher or lower RGR than both older and younger seeds (Supplementary Figure S3). Overall, the RGR was greater for plants growing in dune sand with added compost than in dune sand only (dune sand = 0.0183 ± 0.0002 mg/mg/day, mixed = 0.0197 ± 0.0002 mg/mg/day). In addition, the RGR showed a decreasing trend with increasing seed mass, regardless of population and year of collection (Seed Mass: *F*_1,643_ = 174.16, *P* < 0.0001). Overall, seedlings originating from heavier seeds had a lower RGR compared with those from lighter seeds (Supplementary Figure S4). Finally, the RGR was not significantly affected by any of the climatic variables considered (*P* > 0.05).

## Discussion

Coastal dunes are dynamic and often harsh environments where seed traits play a critical role in plant recruitment, particularly for narrow endemics like *I. littoralis* subsp. *gallaecica*. In this study, we improved our understanding of how seed characteristics such as mass, age, and population origin interact with environmental conditions to influence germination success and early seedling growth. Our results reveal significant variation in seed mass, probability of germination, time taken to germinate, and the RGR of the seedlings across years and populations. These findings highlight the complex interplay between seed age, seed mass, population differences—likely both genetic and environmental—and substrate conditions in determining germination success and subsequent seedling growth.

Seed mass showed interannual and population-level variability, suggesting that both environmental conditions during seed development and potential genetic differentiation among populations contribute to differences in seed size. The maternal environment is known to influence seed provisioning, which makes it plausible that short-term, annual variations in climate may also influence maternal investment, and hence, seed size from year to year (Fenner and Thompson 2005, Donohue 2009). In our study, accumulated precipitation during the seed-development period was indeed associated with lower seed mass, suggesting that wetter years may impose physiological or resource-allocation constraints on mother plants. Higher rainfall could limit photosynthetic efficiency through reduced radiation or could promote growth and shift plants towards producing a larger number of smaller seeds rather than fewer heavier ones (Venable 1992). Averaged across years, populations differed markedly in mean seed mass, with Baldaio producing the smallest seeds, and Doniños and Trece the largest (2.38 ± 0.03 and 2.88 ± 0.03 mg, respectively). Interestingly, these differences align with previous work showing strong genetic differentiation among populations, and very low within-population diversity in most sites, largely due to high selfing rates and the predominance of one or a few genotypes—particularly in Baldaio and Xuño (Lopez et al. 2015). In contrast, Doniños showed high genotypic diversity and lower selfing, which may explain the relatively higher seed mass of its individuals (Lopez et al. 2015). These patterns suggest that, rather than local adaptation, stochastic processes such as genetic drift and inbreeding have shaped the observed differences among populations (Lopez et al. 2015).

The germination timing, proportion of germinated seeds and the RGR of the seedlings also showed significant variation across years and populations. As expected, seed age played a crucial role in germination, with 10- and 9-year-old seeds attaining minimal or no germination. For those seeds collected within the 6 years preceding the study, age also influenced germination, with older seeds exhibiting lower germination rates and longer time to germination. This pattern was particularly pronounced in the populations of Doniños and Ponteceso, suggesting they may be more susceptible to the detrimental effects of ageing. Seed ageing is an irreversible process associated with the progressive accumulation of reactive oxygen species (ROS) and impaired enzymatic activities essential for damage repair (Long et al. 2015, Zhang et al. 2021, Pirredda et al. 2024, Malviya and Gayen 2025). Seed ageing typically manifests as a gradual decline in seed quality over time, which can initially lead to delayed germination and gradually end in the loss of viability, i.e. the loss of the seed’s ability to germinate (Long et al. 2015). Dormancy, defined as the failure of a viable seed to germinate under otherwise favourable conditions (Soppe and Bentsink 2020), can influence both the timing and proportion of germinated seeds. In our study, the observed pattern—i.e. a gradual reduction in germination over time, eventually leading to complete germination failure in the oldest seeds—closely matches the effects of seed ageing rather than dormancy. Although population-level differences in dormancy cannot entirely be ruled out, especially given that dormancy is heritable and varies both among and within species (Baskin and Baskin 2004), evidence from a closely related *Iberodes* species suggests that dormancy is generally short-lived (Neto et al. 2015). Differences in environmental conditions during seed development could have influenced seed traits—including seed quality, dormancy, and viability (Long et al. 2015)—as reflected in the effects of precipitation on seed mass as described above. However, the climatic variables measured—average temperature and accumulated precipitation during the pre- and post-germination periods—did not significantly influence germination, supporting the interpretation that the decline in germination observed here over time primarily reflects intrinsic physiological deterioration. It is also important to note that all seeds were stored under controlled, dry conditions at room temperature prior to the experiments, ensuring that differences in germination were not due to variable storage treatments. Although room-temperature storage may have accelerated seed ageing relative to cold storage, the uniform conditions applied across populations and years help ensure that the observed patterns largely reflect intrinsic seed physiology rather than storage effects.

The RGR of the seedlings varied both among populations and across years, but no consistent pattern of decline with seed age was observed (Supplementary Figure S3). Comparing across years, Baldaio and Ponteceso showed similar RGR across years, indicating little effect of seed age on seedling growth in these populations. In contrast, Doniños, Trece, and Xuño showed significant variation across years, although without a clear directional trend, as intermediate years often had lower or higher RGR than both older and more recent seeds. Although seed deterioration is commonly associated with reduced seedling vigour (Long et al. 2015, Chan et al. 2019), as indicated by reduced root and shoot weight, as well as reduced hypocotyl length (Basra et al. 2003), such effects were not consistently evident in our results.

Seed mass had a significant influence on germination, with heavier seeds generally germinating earlier and reaching higher cumulative germination. These effects varied across populations and years, with the strongest influence observed in seeds collected 4 and 5 years before the experiment, and more moderate effects in younger seeds. Higher germination rates and faster germination for larger seeds have been found in other species (Kolodziejek 2017, Alngiemshy et al. 2020, Connolly 2021, Cevallos et al. 2022), including dune species such as the grass *Leymus arenarius* (Greipsson and Davy 1995) and may be associated with greater seed quality (Ambika et al. 2014). However, the relationship between seed size and germination appears to be complex and not fully understood, with other studies reporting no effects (Tumpa et al. 2021) or an opposite trend (Souza et al. 2014, Yisau et al. 2023). In fact, seed mass did not consistently predict germination success across all populations, with germination of seeds from Doniños and Ponteceso being mostly unaffected by seed mass. These population-specific differences suggest that additional factors, such as genetic differences or differences in seed quality, may override the advantages of a larger seed size for this species.

The climatic variables measured—average temperature and accumulated precipitation during the pre- and post-germination periods—did not significantly affect germination or seedling RGR. However, precipitation during seed development influenced seed mass, with higher rainfall producing lighter seeds. Because seed mass in turn affected both germination (larger seeds germinating earlier and reaching higher cumulative germination) and RGR (lighter seeds producing seedlings with higher RGR), these results suggest indirect influences of maternal environmental conditions, via seed traits, on the germination and seedling growth of *I. littoralis* subsp. *gallaecica*.

Finally, the type of substrate had a significant influence on germination and seedling performance, although the effect on germination was model-dependent. In the model accounting only for fixed effects, substrate was not statistically significant (*P* = 0.072), although the direction of the effect was similar to that observed in the mixed model including population and year as random factors, in which substrate was significant. Cumulative germination was lower, and germination occurred more slowly (shallower curves in Supplementary Figure S2b) in the substrate with added organic material (i.e. the sand with added compost), suggesting that the addition of compost may introduce unfavourable conditions that hinder germination. Soil nutrients have been found to influence germination rates, showing either positive or negative associations depending on the mineral and the plant species considered (Vaughton and Ramsey 2001, Follmer et al. 2021, Kristó et al. 2023). A negative association, as found here, may be related to changes in soil properties beyond nutrients, such as increased water retention, altered pH, or shifts in microbial composition, which could lead to greater seed deterioration (Chee-Sanford et al. 2006). Interestingly, the addition of compost enhanced seedling growth, as indicated by higher RGR values. These findings suggest that while amending dune sand with compost may initially reduce germination rates, such amendments can provide long-term benefits for seedling development, likely through improved nutrient availability and water retention. Nevertheless, the exact soil parameters driving these effects remain unclear, highlighting the need for further research to identify the specific mechanisms underlying these responses.

## Conclusions

Overall, our results underscore the complexity of factors influencing seed germination and early seedling growth of the narrow endemic *I. littoralis* subsp. *gallaecica*. Seed age, population, seed mass, and substrate conditions influence germination success and seedling performance, highlighting the influence of both environmental conditions and potential genetic differences among populations. Our findings emphasise the importance of using fresh seeds for optimal germination and when not possible, selecting the largest seeds from those stored. It also highlights the role of nutrients in supporting early seedling establishment and growth; however, fertilising the soil may not be beneficial if increasing germination rates is the desired outcome. Future studies would benefit from comparing different storage conditions and from a better understanding of how environmental and genetic factors interact to contribute to the observed differences. Understanding these dynamics is essential for conservation and restoration efforts, particularly in heterogeneous environments where seed trait variation may drive differences in establishment success.

## Supplementary Material

plag017_Supplementary_Data

## Data Availability

Raw data and R code are available online at https://doi.org/10.6084/m9.figshare.31820011
