# Supplementary material for: Intraspecific variability in seed mass, germination, and seedling growth in the narrow endemic Iberodes littoralis subsp. gallaecica
Source: AoB Plants. 2026 Apr 6;18(2):plag017. doi: 10.1093/aobpla/plag017 (PMC13093906; doi:10.1093/aobpla/plag017)
Supplement: plag017_Supplementary_Data [file plag017_supplementary_data.pdf]

Supplementary information for:

Sánchez Vilas, Julia, Campoy, Josefina G., Fiuza, Mariana and Retuerto, Rubén.

Intraspecific variability in seed mass, germination, and seedling growth in the narrow endemic *Iberodes littoralis* subsp. *gallaecica*

List of supplementary information in this document:

Table S1. Sample sizes for germinated seeds and seedling relative growth rate (RGR).

Figure S1. Partial regression plots of the effects of climatic variables on seed mass.

Figure S2. Predicted cumulative germination by substrate and pre-germination temperature.

Figure S3. Boxplots of the RGR.

Figure S4. Partial regression plots between seed mass and RGR.

Table S1. Sample size (N) for germinated seeds (G, status =1) and relative growth rate (RGR) of seedlings of *Iberodes littoralis* subsp. *gallaecica*, with data presented for each population and year of collection. Discrepancies in sample size between G and RGR are due to early mortality of seedlings shortly after germination and before harvest.

	**Baldaio**		**Doniños**		**Ponteceso**		**Trece**		**Xuño**	
	**G**	**RGR**	**G**	**RGR**	**G**	**RGR**	**G**	**RGR**	**G**	**RGR**
**2016**	21	19	15	12	2	2	21	20	38	36
**2017**	25	23	5	5	15	13	14	14	27	26
**2018**	30	29	18	16	23	22	34	34	42	42
**2020**	33	31	34	33	34	33	38	37	49	47
**2021**	48	47	46	46	35	35	36	36	47	45

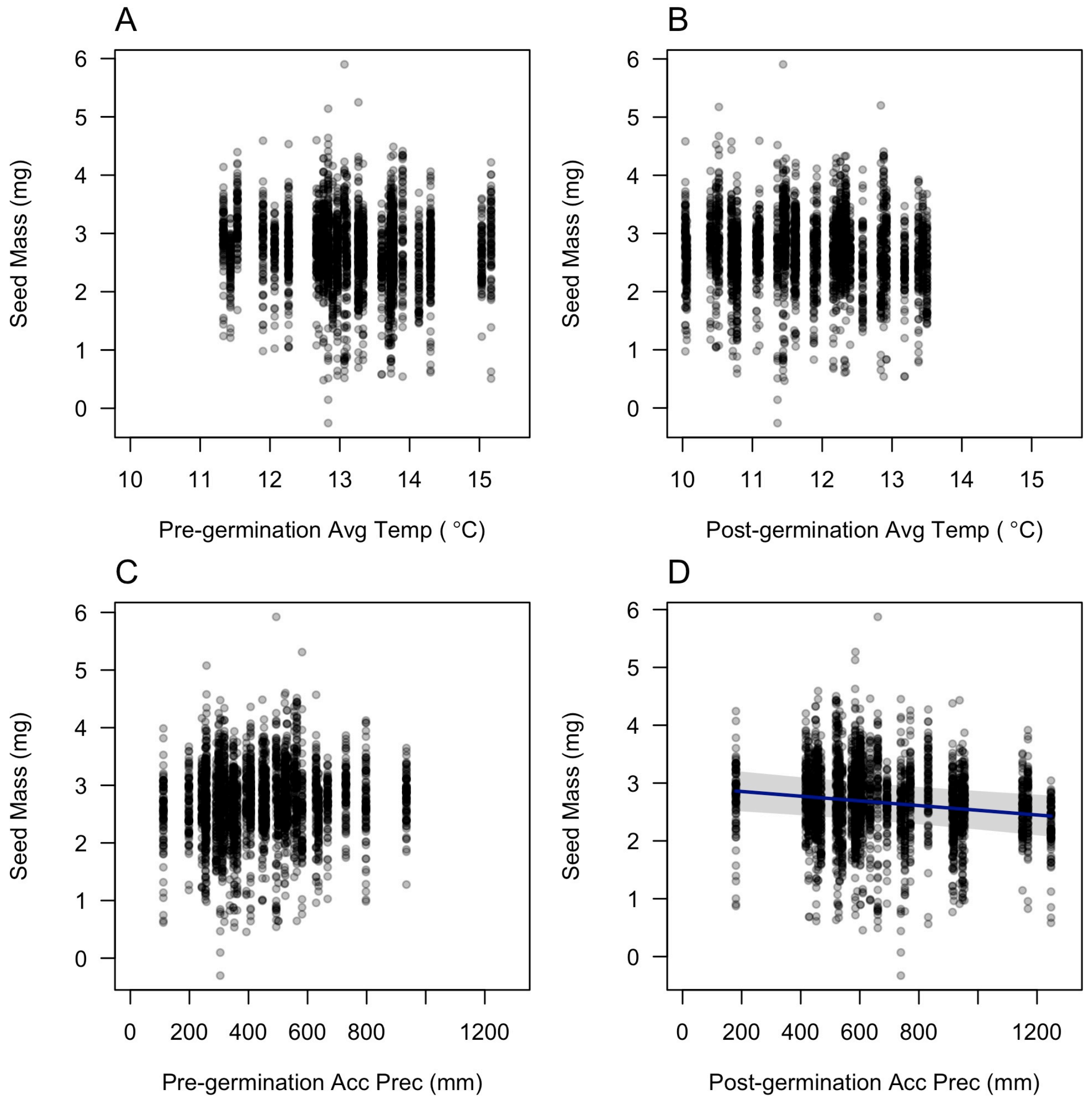

Figure S1. Partial regression plots of the effects of climatic variables on seed mass. Pre-germination Avg Temp = Average temperature (˚C) in the pre-germination period, Pre-germination Acc Prec = Accumulated precipitation (mm) in the pre-germination period, Post-germination Avg Temp = Average temperature (˚C) in the post-germination period, Post-germination Acc Prec = Accumulated precipitation (mm) in the post-germination period. Lines represent model predictions of statistically supported effects.

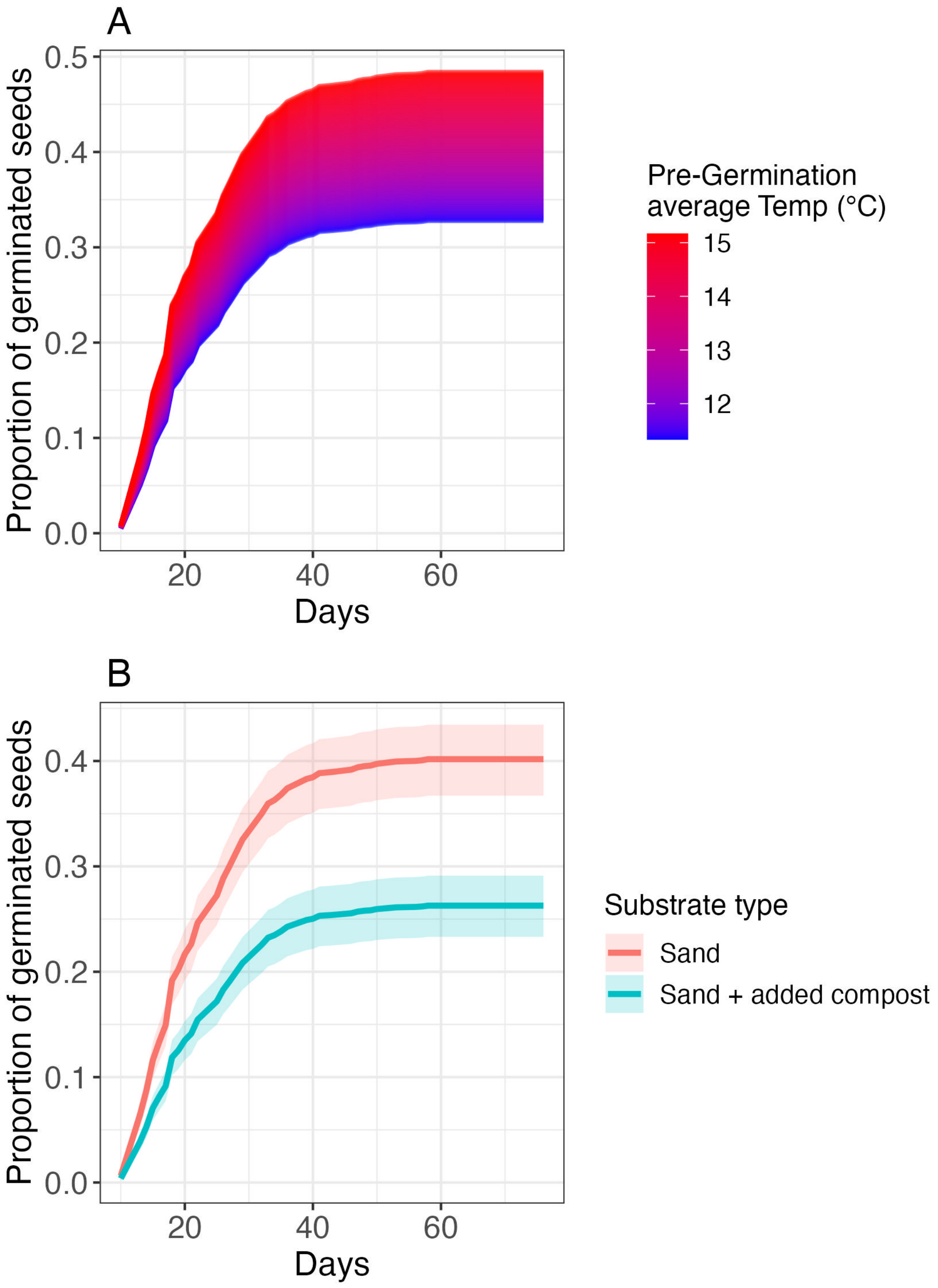

Figure S2. Predicted cumulative germination based on fitted survival models, showing (A) the effects of pre germination (PreG) average temperature – with colour gradient indicating increasing temperatures (°C), and (B) the effects of substrate type (sand, sand + added compost) – with shaded areas representing 95% confidence intervals –. All non-focal predictors were held constant, so plots represent partial effects of the focal predictors on germination dynamics (timing in days and proportion of seeds germinated) of *I. littoralis* subsp. *gallaecica*.

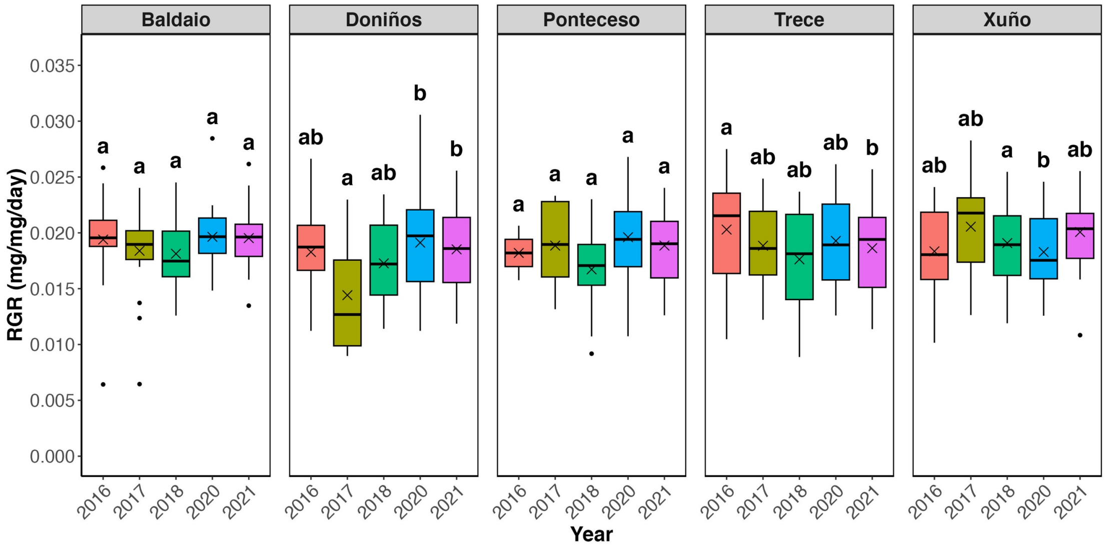

Figure S3. Boxplots showing the distribution of the relative growth rate (RGR) of seedlings of *I. littoralis* subsp. *gallaecica* sourced from different populations and collected in different years [see Table S1 for number of replicates]. Within each box, horizontal lines denote median values, crosses denote mean values; boxes extend from the 25th to the 75th percentile of each group's distribution of values; whiskers denote adjacent values (i.e., the most extreme values within 1.5 interquartile range of the 25th and 75th percentile of each group); dots denote observations outside the range of adjacent values. Different letters above each box indicate significant differences between years within the same population ($P < 0.05$).

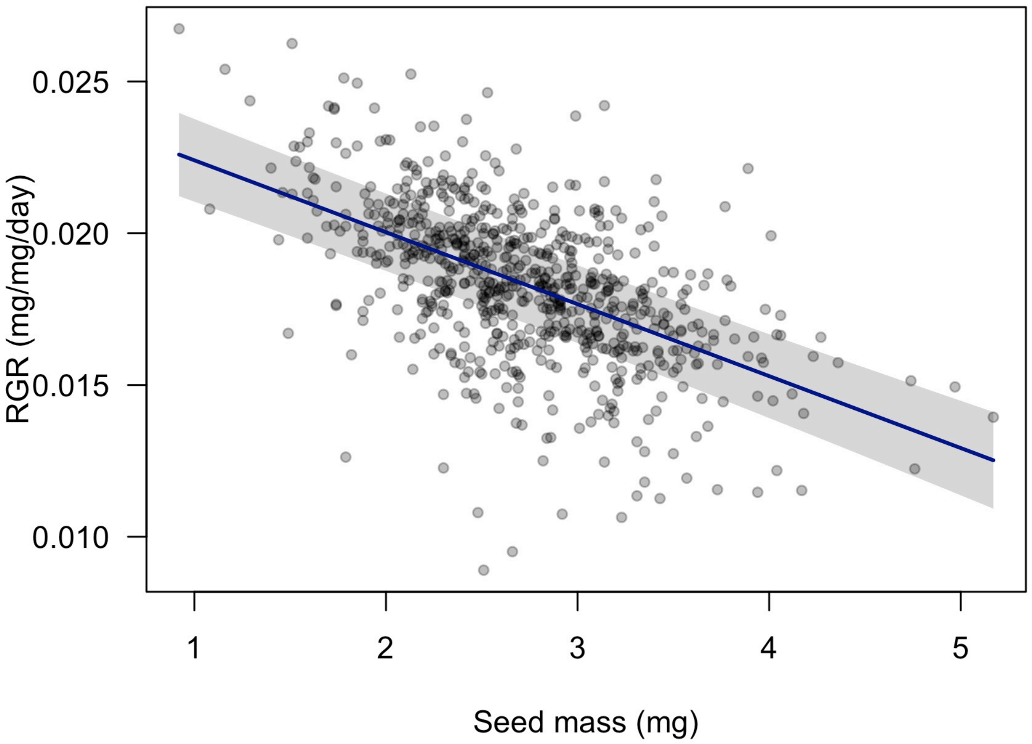

Figure S4. Partial regression plot showing the relationship between seed mass (mg) and relative growth rate (RGR; mg mg^{-1} day^{-1}) of seedlings. Points represent partial residuals, illustrating the effect of seed mass after accounting for other variables in the model. The solid line shows the fitted values from the linear mixed-effects model, and the shaded area represents the 95% confidence interval.
